# Effect of Neck Muscle Fatigue on Hand Muscle Motor Performance and Early Somatosensory Evoked Potentials

**DOI:** 10.3390/brainsci11111481

**Published:** 2021-11-09

**Authors:** Mahboobeh Zabihhosseinian, Paul Yielder, Rufeyda Wise, Michael Holmes, Bernadette Murphy

**Affiliations:** 1 Faculty of Health Sciences, University of Ontario Institute of Technology, 2000 Simcoe St. North, Oshawa, ON L1G 0C5, Canada; Mahboobeh.Zabihhosseinian@ontariotechu.net (M.Z.); Paul.yielder@ontariotechu.ca (P.Y.); Rufeyda.wise@ontariotechu.net (R.W.); 2 Department of Kinesiology, Brock University, 1812 Sir Isaac Brock Way, St. Catharines, ON L2S 3A1, Canada; mholmes2@brocku.ca

**Keywords:** somatosensory evoked potentials, motor skill acquisition, sensorimotor integration, cervical extensor muscle fatigue

## Abstract

Even on pain free days, recurrent neck pain alters sensorimotor integration (SMI) measured via somatosensory evoked potentials (SEPs). Neck muscle fatigue decreases upper limb proprioception, and thus may interfere with upper limb motor task acquisition and SMI. This study aimed to determine the effect of cervical extensor muscle (CEM) fatigue on upper limb motor acquisition and retention; and SMI, measured via early SEPs. Twenty-four healthy right-handed individuals were randomly assigned to control or CEM fatigue. Baseline SEPs were elicited via median nerve stimulation at the wrist. Participants then lay prone on a padded table. The fatigue group supported a 2 kg weight until they could no longer maintain the position. The control group rested their neck in neutral for 5 min. Participants completed pre- and post-motor skill acquisition while seated, SEPs were again collected. Task retention was measured 24 h later. Accuracy improved post acquisition and at retention for both groups (*p* < 0.001), with controls outperforming the fatigue group (*p* < 0.05). The fatigue group had significantly greater increases in the N24 (*p* = 0.017) and N30 (*p* = 0.007) SEP peaks. CEM fatigue impaired upper limb motor learning outcomes in conjunction with differential changes in SEP peak amplitudes related to SMI.

## 1. Introduction

Sensorimotor integration (SMI) refers to the ability of the central nervous system (CNS) to integrate sensory information from the environment and formulate appropriate motor outputs [1]. Short latency somatosensory evoked potentials (SEPs) (<30 ms) have been used to measure changes in SMI in various experimental paradigms involving motor skill acquisition tasks. Past studies utilizing SEPs have shown differential changes following motor skill acquisition in subclinical neck pain populations tested on pain-free days [2], suggesting that altered sensory input from the neck impacts upper limb SMI. Motor skill acquisition tasks have demonstrated morphological or functional changes in neuronal or network connections within cortical and subcortical areas as well as distinct interactions that occur between cortico-striatal and cortico-cerebellar systems [3,4], and any changes in this neural network can alter CNS function, outlasting the period of the altered afferent input itself [5]. The cerebellum plays an essential role in motor learning with activation in response to a period of fast motor learning [6], and reduction in activation once the task is well learned [7]. The cerebellum can modulate sensory inputs that create and modify motor responses based on expected sensory outputs [8], thereby directly influencing neural pathways between the cerebellum and the sensorimotor cortex. Excitability changes in these pathways can be measured using short latency SEPs [9]. 

Neck sensory receptors have central and reflex connections to the vestibular, visual and sensorimotor integration areas of the CNS [10]. Neck afferent inputs are important in coordination of movement and like the vestibular system, they have numerous projections to the cerebellar cortex [11]. Previous animal studies have been shown electrical stimulation of the neck afferent fibers at the C2 dorsal root ganglion resulted in electrical activity in lobules V and VI of the anterior lobe of the cerebellum, reaching the cerebellum by way of both mossy and climbing fibers [11]. Neck afferent flow to the cerebellum converges with projections from other sensory systems as well as extraocular muscle afferents, trigeminal afferent fibers, and forelimb and hind limb input [11]. Neck somatosensory input plays a role similar to the vestibular system. Neck motoneurons have polysynaptic networks within the superior colliculus [12], and monosynaptic excitation or inhibition of neck motoneurons can be initiated from the vestibular nuclei [13]. A study in squirrel monkeys found that vestibular and neck afferent inputs converge with specific patterns on the cerebellar interpositus nucleus [14]. Two distinct populations of neurons were found, one conveying head position in space. The second showed exact matching of vestibular and proprioceptive stimuli, even when the stimuli were complex, activating vestibular sensory receptors and involving neck rotation and lateral flexion [14]. These neurons effectively encode body motion in space, which is critical for accurate control of upper limb movement. Neck afferent inputs can be impaired by trauma, functional damage of sensory receptors, changes in muscle spindle sensitivity, and pain at many levels of the nervous system, all of which can alter the integration, timing and tuning of sensorimotor control [10]. 

Neck muscles contain a high density of sensory receptors [15], therefore altered sensory input due to neck pain [2], or fatigue [16], can affect sensory feedback from the neck muscles to the CNS, resulting in impaired upper limb motor control [2], with functional neck impairment linked to altered muscle recruitment patterns known to decrease postural control [17]. Short latency SEPs represent neural processing at specific neural generators. The source of different SEP peaks has been determined during neurosurgery, lesion studies and more recently, source localization [18,19,20]. Neck pain has been shown to affect SEP measurements related to SMI, resulting in maladaptive neural plastic changes [2], that improved following neck treatment [18]. Similarly it is known that individuals with recurrent neck pain demonstrate differential SEP peak changes relative to controls following a motor learning task in SEP peaks related to cerebellar processing and SMI [2], indicating that altered neck input can impact early somatosensory processing as measured by short latency SEPs.

In order to control body movement in three-dimensional space, an awareness of the integrated neural representation of body parts (the body schema) and their virtual motion in space and time in relation to the environment is required to pursue behavioral goals [19]. In order to plan motor commands for a movement, the CNS relies on both feedforward and feedback models, which are the result of coordination of both visually observed consequences of the motor command, and its proprioceptive feedback [20]. An accurate body schema is critical for generating accurate and coordinated movement [21]. The internal body schema updates the connection between the motor commands and the expected sensory outputs, and any altered sensory inputs to the cerebellum from the periphery can change the inputs from the cerebellum to the motor cortex, leading to maladaptive plasticity [22].

Previous experiments suggests that the internal body schema may be impaired in neck pain [23] and also after neck muscle fatigue [24], due to altered sensory input from the neck. Neck muscle fatigue can also change upper limb proprioception, with reduced ability to accurately replicate elbow and forearm positions [16]. Additionally, passive head and neck movement, as well as an external vibration to the neck muscles can influence the accuracy of elbow and forearm proprioception [25]. These studies demonstrated that it was the altered neck afferent input, rather than vestibular changes that influenced spatial awareness of the limbs [26], thereby indicating that sensory input from the neck impacts the internal body schema in humans, with likely implications for SMI and upper limb motor control.

Fatigue is a complex phenomenon due to peripheral changes of the muscle [27], as well as decreased neural drive to motoneurons (central fatigue) [27]. Cervical extensor muscle (CEM) fatigue, has been shown to alter upper limb proprioception [16], as well as performance on tasks requiring accurate upper limb proprioception [24]. CEM fatigue leads to altered afferent input from the neck to the CNS for several minutes following cessation of a fatiguing task, and is known to impact the way that the brain controls the neck following fatigue [16]. We recently used a dual pulse transcranial magnetic stimulation protocol and showed that fatigue prior to motor acquisition decreased disinhibition of the cerebellum to M1 pathway and lead to worse performance at acquisition and retention [28]. Given these changes in motor pathways, it seems critical to investigate the effect of CEM fatigue on sensory processing in response to motor skill acquisition. Short latency SEPs provide a means to do this. This study explored the role that CEM fatigue has on cerebellar and SMI pathways in response to novel motor skill acquisition. It was hypothesized that a novel motor skill acquisition tracing task, when performed following CEM fatigue, would result in differential changes in short latency SEP peaks previously shown to change in response to motor skill acquisition (i.e. using dipole source localization, the neural generator of the N24 peak was identified near the wall of the central sulcus in the area 3b pathway between the cerebellum and the SI. Reduction or absence of N24 peak on those with cerebellar lesions indicates that N24 peak is directly linked to the integrity of the cerebellum [29]. The N30 peak originates in the post-central cortical regions [30], and it is a complex subcortical and cortical loop connecting the basal ganglia, thalamus, pre-motor areas, and primary motor cortex [31,32]. Therefore, the N30 SEP peak has multiple neural generators sub serving motor control and sensorimotor integration [33], coinciding with diminished performance accuracy both post- motor skill acquisition, and subsequent task retention, relative to healthy controls. Based on previous motor skill acquisition studies, the spinal N11 and N13 peaks were not expected to change, but were included to ensure there were no spinal changes.

## 2. Methods

### 2.1. Ethical Approval

Written and verbal informed consent was obtained and the study was approved by the Ontario Tech University Research Ethics Board (REB). This study was carried according to the ethical standards set out by the Declaration of Helsinki statutes governing research on human subjects.

### 2.2. Participants

GPOWER statistical software indicates that for a large effect size (0.4) with an alpha of 0.05 and a power of 0.95, 12 participants are needed for each group in a pre-post experimental design [34]. Two groups of 12 healthy individuals with no known neurological conditions (mean age: 21.25 ± 0.85) were randomly assigned to either a fatigue (6 males, 6 females) or a control (6 males, 6 females) group. An Excel random number generator was used to randomly give each male and female participant a number from 1 to 12, with coding to ensure 6 even and 6 odd numbers per randomization. The odd numbers in each group were assigned to the fatigue group and the even numbers were assigned to control group, thus ensuring equal proportions of males and females in each group. Due to possible sex-related differences in neuromuscular performance and fatigability between men and women [35] and motor skill acquisition, efforts were made to ensure equal proportions of males and females in each group.

### 2.3. Inclusion and Exclusion Criteria

As cortical plasticity has been linked with age, we aimed to test young, healthy right-handed participants. Right handed individuals were tested due to known differences in cortical excitability between dominant and non-dominant limbs in response to performing a novel motor tracing task [36]. All participants reported themselves as right-hand dominant, and it was also confirmed by the Edinburgh Handedness Inventory (EHI) self-report questionnaire [37]. Participants were free from chronic or recurrent neck, shoulder, elbow, and wrist pain and injury and had full pain-free neck and shoulder range of motion for at least 3 months prior to data collection. The Neck Disability Index (NDI) self-report questionnaire [38] was administered to confirm the absence of neck pain (scores of 0–4/50), as pain is known to alter some of the dependent measures of this current study. Each participant completed a confidential health history in order to identify any exclusionary medical conditions that could influence normal somatosensation including recent cervico-thoracic injury, neurological conditions, current use of neuroactive or pain medication, history of epilepsy, heart disease, presence of metal fragments in the head, upper body or eye, and pregnancy. All participant’s age, NDI and EHI data are found in Table 1.

### 2.4. Experimental Protocol

**Figure 1:**
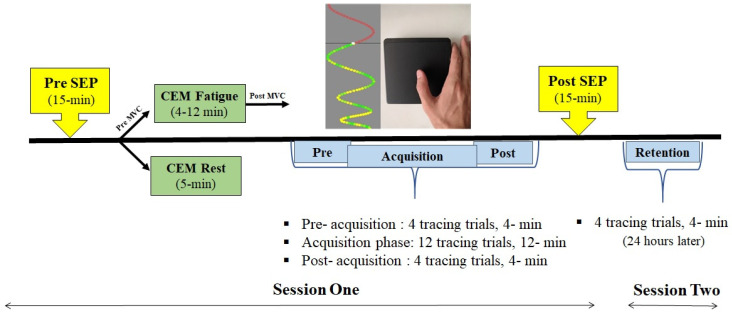
Experimental protocol: The first session started by obtaining the pre-SEP (baseline) measurements through the stimulation of the median nerve and then participants completed either cervical extensor muscle (CEM) rest or fatigue, depending on the group. Each participant completed the motor skill acquisition-tracing task, then post-SEP measurements were obtained. The second session measured retention 24 h after using the same pre- and post-motor skill acquisition task performed in session one. For motor skill acquisition, a tracing task of continuous sinusoidal waves move vertically down a monitor and participants attempted to copy each dot as it passed the black horizontal axis. Color coding of the dots show trace accuracy feedback with green representing a perfect trace and yellow indicating error.

This study was a between group experimental design, comparing the effects of muscle fatigue and motor skill acquisition on a group with CEM fatigue to a control group. All participants were required to attend two sessions, 24 h apart. After applying all instrumentation, the first session started by obtaining the pre-SEP (baseline) measurements (total 15-min) through the stimulation of the median nerve with slow rate 2.47 (5-min) and fast rate 4.98 Hz (10-min) (described below). Participants then completed either CEM rest or fatigue, depending on the group. The fatigue group performed one CEM maximal voluntary contraction (MVC) held for 3 s, followed by a CEM fatigue protocol (until failure), while the control group rested their head (5-min), while lying prone on a table. Each participant completed the motor skill acquisition task consisting of pre-acquisition (4 tracing trials, 4-min), acquisition phase (twelve tracing trials, 12-min), and post-acquisition (four tracing trials, 3-min). Then post-SEP measurements were obtained (15-min). The second session (24 h later) measured retention using the same pre- and post- motor skill acquisition task performed in session one (4-min). During the second retention session, which measures the later consolidation stage of motor learning, SEPs were not collected, as past work suggests that the majority of excitability changes in cortico-cerebellar pathways occur on day one during the early stage of motor learning [39]. A schematic of the protocol design can be seen in Figure 1. 

### 2.5. SEP Recording Parameters

**Figure 2:**
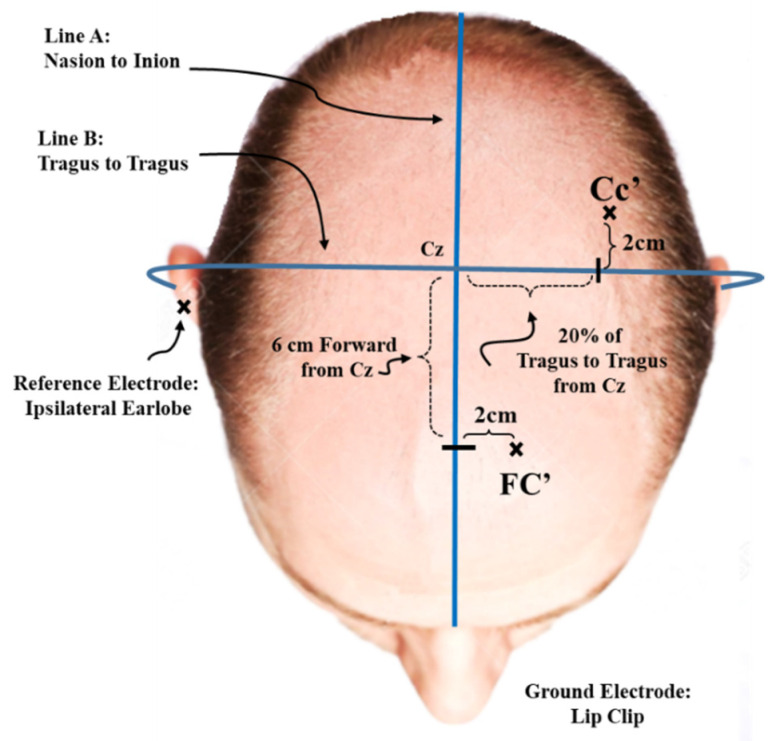
Schematic of cephalic site recording EEG electrode placement: According to the EEG adhesive conductive paste (type TEN20) international EEG system, the cortical locations placed contralateral to the site of stimulation: on parietal site (CC’) (20% of the subject’s tragus to tragus measurement and 2 cm posterior to contralateral to vertex or Cz), and frontal site (Fc’) (6 cm anterior and 2 cm contralateral to Cz), and with an ipsilateral earlobe reference.

SEP recording electrodes were placed according to the International Federation of Clinical Neurophysiologists (IFCN) guidelines [40]. Electroencephalography (EEG) electrodes were placed according to the recommendations with Grass Technologies EEG adhesive conducting paste (type TEN20) international EEG system, with cortical locations placed contralateral to the site of stimulation and an ipsilateral earlobe reference [33]. Surface EMG electrodes (Ag-AgCl, Meditate, conductive adhesive hydrogel) were placed on the ipsilateral brachial plexus (Erb’s point) for upper extremity SEPs [33], over C5 spinous process (Cv5), and the anterior neck or trachea (Supraglottic region on the midline) [33]. The cephalic site recoding EEG electrodes were placed on parietal site (CC’) (20% of the subject’s tragus to tragus measurement and 2 cm posterior to contralateral to vertex or Cz), and frontal site (Fc’) (6 cm anterior and 2 cm contralateral to Cz) [33] (Figure 2). Prior to each electrode placement, the sites were prepared and cleaned with abrasive pads and alcohol swabs. To minimize the electrical artifact produced by the stimulation, the ground electrode was placed between the stimulation site and the recording electrodes, in the mouth. The two cephalic site electrodes and the ground electrode were 1.8 m long traditional leads (10 mm disc, 2 mm hole gold cup EEG electrodes, Grass Technologies; Astro-Med; Subsidiary, Rockland, MA; impedance < 5 kΩ). The C5 spinous process was referenced to the trachea, while all other electrodes were referenced to the ipsilateral earlobe, as this minimizes the stimulus artifact.

The amplitudes and latencies of the following short-latency SEP components were identified and analyzed: the peripheral N9, the spinal N11 and N13, and the far-field N18 (P14–N18 complex), the parietal N20 (P14–N20 complex), and P25 (N20–P25 complex), the frontal N24 (P22–N24 complex), and the frontal N30 (P22–N30 complex). The N9 SEP peak is recorded at Erb’s point, over the brachial plexus. The N9 peak amplitude provides a way to ensure that the peripheral afferent nerve volley is stable, so that changes in subsequent SEP peaks can then be attributed to changes in activity at the relevant neural generator. The N11 and N13 SEP peaks can be recorded over the 5th (Cv5). The N11 indicates the afferent volley arriving to the spinal cord as it begins to travel to the cuneate nucleus [41]. The N13 peak is thought to reflect activity in dorsal horn interneurons [42]. The N18 SEP peak is recorded from a contralateral frontal cephalic site (Fc’) [33]. There is clinical evidence that the N18 peak is generated in the brain stem, up to the level of the midbrain-pontine region [42,43] between the lower medulla and midbrain-pontine region (specifically nuclei in the dorsal column medial lemniscus and accessory inferior olives) [42,43]. The contralateral parietal N20 SEP peak is recorded from 2 cm posterior to 20% of contralateral central scalp site which is referred to as Cc’. The N20 reflects the earliest cortical processing in the primary somatosensory cortex (SI), representing activity of a dipolar generator in Brodmann’s area 3b situated in the posterior bank of the rolandic fissure [40,44]. The P25 peak is recorded from 2 cm posterior to the 20% contralateral central scalp site (Cc’) with a reference electrode placed on the ipsilateral earlobe. The origin of N20- P25 is still debated and a general agreement has been reached on the hypothesis that this component most likely represent a tangential dipole across the central fissure and arises from the posterior bank of the central fissure, area 3b [45] and current sources at the primary somatosensory cortex [45]. The N24 is recorded from a contralateral frontal cephalic site (Fc’) from faster sampling rate and is identified near the wall of the central sulcus in the area 3b pathway between the cerebellum and the SI, close to the location of the N20 peak [46]. The N30 is recorded from a contralateral frontal cephalic site (Fc’) from slower sampling rate and originates in the post-central cortical regions [29,33], and it is a complex subcortical and cortical loop connecting the basal ganglia, thalamus, pre-motor areas, and primary motor cortex [31,32,33]. Using novel imaging technology, swLORETA (standardized weighted low-resolution brain electromagnetic tomography), the motor, premotor, and prefrontal cortex networks are primarily involved in generating the peak of N30 SEP [47]. The N30 peak is generated by oscillating and phasic generators in the frontal cortex. The beta-gamma power increase and phase-locking generation of N30 are located pre-centrally. Therefore, the N30 SEP peak reflects SMI [33] and has multiple neural generators.

SEPs were recorded at two different sampling rates to enable optimal conditions to record both the N24 and N30 SEP peak complexes. The N24 SEP peak is usually detected as a notch that remains in upward slope of the N30 SEP peak [48]. The slow rate, 2.47 Hz (405 ISI) (5-min), does not lead to N30 SEP peak attenuation [49], while the fast rate, 4.98 Hz (201 ISI) (10-min), attenuates the N30 SEP peak, allowing for a more clearly defined N24 SEP peak, which can be more accurately measured [49]. During the SEPs data collection, participants were in a quiet room and seated in a comfortable, but rigid office chair with their eyes open. 

### 2.6. Stimulation Parameters

Stimuli consisted of electrical square pulses, 200 µs in duration, delivered at constant intensity at frequencies of both 2.47 Hz and 4.98 Hz through Ag/AgCl EMG conductive adhesive surface electrodes (Meditrace™ 130, Kendall, and Mansfield, MA, USA) (impedance <5 kΩ ) connected to the cathode and anode of the stimulator (Digitimer DS7A constant current, Welwyn Garden city, UK). These electrodes were placed on the skin overlying the median nerve of the right wrist, between the tendons of flexor pollicis longus and palmaris longus. The cathode, was placed 2 cm proximal to the wrist crease and the anode on the wrist crease in order to prevent anodal block [50]. This position allows for movement of the Abductor Pollicis Brevis (APB) muscle, through stimulation of the motor branches of the median nerve that mainly innervates this muscle. SEP peak amplitudes were measured from the averaged 1500 sweeps of the waveforms.

The intensity of the electrical stimuli were delivered until motor threshold was reached for each participant, which was defined as the lowest possible stimulation intensity that elicited a visible and constant thumb twitch (approximately 10 mm) of the APB muscle. At this stimulus intensity, all SEP components peaking before 50 ms post stimulus can reach their maximal amplitude [50]. Stimulating above the motor threshold ensured that the fastest conducting group 1a afferents, responsible for much of the short latency SEPs, are being stimulated [51]. The activity in group 1a muscle afferents project to the cerebral cortex following the median nerve stimulation [51]. 

### 2.7. Motor Skill Acquisition Tracing Task Parameters

The motor skill acquisition-tracing task was run and analyzed through a custom-written (C++) Leap Motion software tool (Leap Motion, Inc., San Francisco, CA, USA). The traces were formed by a series of continuous sinusoidal-pattern waves composed of colored dots, which moved vertically down a monitor, while the participant attempted to trace each dot as it passed the horizontal axis. This horizontal axis has a single dot with the same radius as the dots composing the sinusoidal-pattern waves. Four unique preselected sinusoidal-pattern waves were designed to change in complexity through the variation and randomization of both the frequency and amplitude of the sinusoid. This ensured continuous learning, allowed for unpredictability throughout the duration of the trace [52] and utilized a protocol known not to result in hand muscle fatigue [53]. Each trial required the participant to constantly adjust velocity and abduction/adduction range of motion of the thumb, involving a sweep from left to right, utilizing the abductor pollicis brevis (APB) muscle. Color-coding of the dots provided trace accuracy feedback with green representing a perfect trace and yellow some error. The sinusoidal-pattern waves are close together at the beginning and get wider at the end of each trial (Figure 1). 

The participants were seated upright in front of an adjustable table, which had a monitor that was presenting the sinusoidal-pattern waves. To ensure the stability of the arm, to isolate the thumb movement during task performance, and to prevent shoulder fatigue, the participant’s forearms were rested atop adjustable armrests. The height of the armrest was set to allow the shoulder to remain in a neutral position and the table’s platform was level with the armrest. The position and direction of the hand was marked to ensure a consistent position during median nerve stimulation. The thumb was positioned on an external wireless touchpad (Logitech, Inc., Fremont, CA, USA) to perform the tracing task. No obvious delay time occurred between the participant’s movements on the external tracing pad and the movement of the traces observed on the monitor. At each training session, the participant completed three blocks of training for the pre-acquisition, post-acquisition, and retention trials, with each of the four trace conditions performed once in approximately 3-min time duration. For the acquisition phase, each version was performed three times for a total of 12 traces, taking approximately 10-min prior to the start of each block for each participant, the order of the task version was pseudo-randomized to control for possible order effects in completing the different tasks [36]. Previous work using the same tracing tasks has shown that the simplest trace was easy to trace for all participants and the hardest was challenging [36]. Motor error was analyzed by determining the average distance of the attempted trace from the original template trace. Improvement in performance refers to a decrease in the percentage of error throughout the task. 

### 2.8. CEM Surface EMG

Muscle activity was measured bilaterally from the CEM at the level of the C4/C5 spinous process using surface EMG (Meditrace™ 130, Kendall, and Mansfield, MA, USA). Following shaving and cleansing the skin with an isopropyl alcohol swab, surface EMG electrodes were placed bilaterally over the muscle belly of CEM, in line with fiber orientation, and 2 cm lateral to the space between the spinous processes of C4 and C5 and a ground electrode was over the right clavicle. EMG activity and force were measured through Lab Chart 7™ (AD Instruments, Sydney, Australia).

### 2.9. CEM Fatigue Protocol

**Figure 3:**
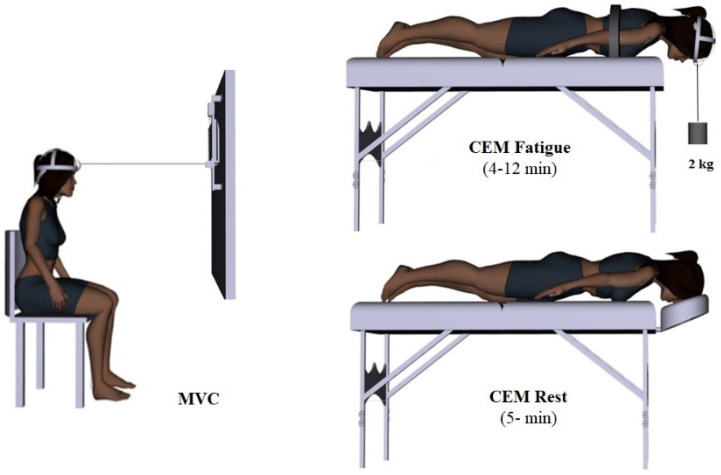
Left, maximum voluntary contraction set up. Isometric head/neck extension against a wall-mounted force transducer with the angle between the cable attached to the force transducer and the participant’s head remained at 90°. Each participant performed one MVC, held for 3 s. Right, submaximal CEM fatigue/rest protocol. Top, participant performing fatigue protocol. Bottom, control participant laying prone on the table for 5 min. The figures were created using Santos Pro software (SantosHuman, Inc., Coralville, IA, USA).

Prior to CEM fatigue, participants performed a neck muscle warm up with 10 range of motion repetitions, consisting of neck flexion, extension, and lateral flexion and rotation to both sides. Next, one maximal voluntary contraction (MVC) of the CEM was performed pre- and one immediately post- fatigue interventions for fatigue group. In order to maximize motivation [27], each MVC was accompanied by verbal encouragement from the experimenters to exert a maximum head extension force against a wall-mounted adjustable force transducer (Model: BG 500, Mark-10 Corporation, NY, USA) that was attached via a cable to a Nexgen™ ergonomic strap fixed to the participant’s head (Figure 3). The angles between the cable attached to the force transducer and the participant’s head were maintained at 90°. The cable was horizontal and participants were instructed to pull directly in line with the cable, while maintaining an upright and consistent posture. Participants were seated on a chair with no upper thoracic or cervical support, with the hips and knees at 90°, feet crossed on the floor and arms crossed on their lap to prevent bracing and to minimize any additional leverage such that the created force were mostly from the cervical extensors [16]. Verbal encouragement was given by the investigators and each participant performed one MVC, held for 3 s. Peak force was determined from the MVC and then each participant performed the CEM fatigue protocol. 

The CEM fatigue protocol was first described by Edmondston et al. [30], which has been shown to have a good test–retest reliability. Participants lay prone with their head over the edge of a table, initially supported by a head support. To counter-support the upper thoracic spine, a strap was fixed around the thorax at the level of T6 to isolate the CEM. A Velcro strap was fixed around the head and an inclinometer (Carpi digital angle gauge ^TM^, accuracy ±0.1°, resolution 0.1°, CP20005, Carpi tools, Pomona, CA, USA) was placed superior to the right ear to measure sagittal head position. Before starting the fatigue protocol, the head and neck were placed on the headrest in a comfortable neutral position and the test began by removing the head support. A 2 kg weight hung from the head and participants held the head in a horizontal position, with the chin retracted, until they could no longer maintain a neural head posture (Figure 3). The body position was monitored during the test to detect any upper limb movements and the protocol was terminated when participants even with encouragement could not maintain the head posture due to discomfort or pain or neck muscle exhaustion, or if the horizontal position of the head changed from initial position by more than 5 degrees towards the floor for more than 5 s.

### 2.10. CEM Rest Protocol

The control participants lay prone for 5 min on a padded table with their head supported on a headrest over the end of the table and arms alongside with their trunk (Figure 3).

## 3. Data Processing

The outcome measures of this study were analyzed separately, which included CEM fatigue, motor performance on the tracing task pre and post-acquisition and retention, and the amplitude of the early SEP peaks pre and post motor skill acquisition.

Maximum peak force prior to CEM fatigue, mean power frequency (MNF) and root mean square (RMS) of the CEM sEMG during the first and last 10 s of the fatigue protocol were calculated using Lab Chart 8™ (AD Instruments, Sydney, Australia) [16]. Because all participants were right hand dominant, it was possible there might be differences in fatigability between the right and left CEM; therefore, fatigue indices were calculated for both sides.

The sinusoidal tracing task data were exported to Excel™ (Microsoft Office version 16). Accuracy of the motor skill acquisition tracing task data represented as percent error determined as the average distance of the attempted trace from the template trace, where 100% error represented one dot width (6.62 mm), in distance away from the template. For each participant, percent errors were averaged for each trial in all test conditions, including the pre-acquisition, post-acquisition and retention tests.

The SEP signals were amplified (gain of 10,000), filtered (0.2–1000 Hz), and saved on a laboratory computer for further retrieval using a configuration written in Signal software (Version 4.08, Cambridge Electronic Design, Cambridge, UK). The amplitude of a SEP peak represents the volume of activity of the neural structure(s) generating that peak, and any fluctuation detected in that peak amplitude represent alterations in the activity of the neural generator(s) [54]. The latency of the SEP peaks is the conduction time between the site of stimulation and the onset of the waveform for a given neural generator, and any variation in this peak latency represent alteration in neural transmission [44]. The two most commonly measured variables in SEPs studies are the amplitude microvolts (µV) and latency (ms) of the peaks representing activity of different neural generators [55]. The amplitudes of the SEP peaks were measured from the peak of interest to the earlier or later peak of opposite deflection [40]. SEP peak latencies were recorded from the onset time of the stimulation to the maximal peak deviation or between each of the SEP peak components. SEP peaks are waveform components named based on their deviation direction and latency. The ionic current flow across the cell membranes of active neural structures result in potential differences in voltage [56]. Along the transmission pathway, the area of the cortex or spinal cord producing these positive or negative potentials is named a neural generator [44]. SEP peak amplitudes can be used to measure changes in the activity of underlying neural generators, with greater peak amplitudes observed when scalp-recording electrodes are closer to the neural generator responsible for that peak [44,56]. 

SEP peak amplitudes were measured in both pre- and post-motor skill acquisition task and latencies were determined for any variations in processing time or speed subsequent to the post-motor skill acquisition performance. Stable afferent input is essential in order to attribute any subsequent changes in SEP peak amplitudes to learning-induced plasticity. The N9 peak, measured over the brachial plexus, provides an indicator of a stable and consistent peripheral nerve volley (N9 SEP peak). Therefore, the N9 SEP peak had to be within ±20% between pre- and post-intervention trials in order to include a participant’s SEPs data in the study. This percent variation would indicate any observed potential changes that the SEP peaks have on central generators and were due to motor skill acquisition or CEM fatigue interventions and not due to alteration in the incoming afferent volley. As two separate groups (control-fatigue) were being compared to each other, data from SEP peak-to-peak amplitudes from pre- and post-motor skill acquisition task were normalized to the baseline values, being expressed as a proportion of the baseline, to account for inter-participant baseline variability and to allow for between-group comparisons.

## 4. Statistical Analysis

### 4.1. Baseline Differences between Groups

Independent samples *t*-tests were used to determine if there were any baseline differences between the fatigue and control groups in age, NDI, and EHI scores.

Time to task failure: Separate independent samples *t*-tests compared the MVC force level (pre vs. post fatigue), and the time to task failure between male and female participants. Myoelectric fatigue was assessed by separate 2 × 2 repeated measures analyses of variance (ANOVAs) to examine MNF and RMS with fatigue (pre vs. post) as the repeated measure, and CEM (right and left) as the between subject factor.

### 4.2. Behavioral Data

The accuracy of the motor skill acquisition-tracing task, which was the average percent error for pre-acquisition, post-acquisition and retention were compared between the fatigue and the control group. The accuracy data was normalized to each individual’s baseline value, and a 2 × 3 repeated measures ANOVA with TIME (pre-acquisition, post-acquisition, and retention) as the repeated measure and GROUP (control vs. fatigue) as the between factor was performed on the normalized data, in order to enable comparison of the relative improvements in performance between groups. Post-hoc ANOVA (Bonferroni correction) were used to compare the pre- and post-acquisition and retention between the groups.

### 4.3. Neurophysiological Data

The interactive effect of fatigue and the motor skill acquisition tracing task on each SEP peak amplitude, was tested with a 2 × 2 mixed-design repeated measures ANOVA with time (pre-acquisition vs. post-acquisition) as the repeated measure and group (control vs. fatigue) as the between subjects factor for each SEP peak.

Statistical significance was set at *p* ≤ 0.05 for all analysis (SPSS v.24, IBM Corporation, Armonk, NY, USA). All numeric values are expressed as mean ± standard deviation (SD).

## 5. Results

### 5.1. Participants

Demographics of the control and fatigue groups are summarized in (Table 1). There were no significant differences in demographics between groups. The mean baseline NDI and EHI scores for both fatigue and control group confirmed that all participants were free of neck pain and right-hand dominant. None of the participants in the fatigue group stopped the fatigue protocol due to neck or upper body pain.

### 5.2. Fatigue Data

There were significant differences between pre- and post-MVCs (*p* = 0.0001), with a decrease in force from the pre fatigue-MVC (131.45 ± 39.83 N), to the immediately post fatigue-MVC (95.16 ± 17.50) in the fatigue group. The mean pre MVC for males and females was 163.24 ± 27.98 and 99.67 ± 16.83 N, and post MVC was 109.08 ± 6.55 and 81.25 ± 12.88 N, respectively. The mean contraction time to task failure was 6.29 ± 2.84 min, with no significant differences between sex (*p* = 0.457) (Table 1).

**Figure 4:**
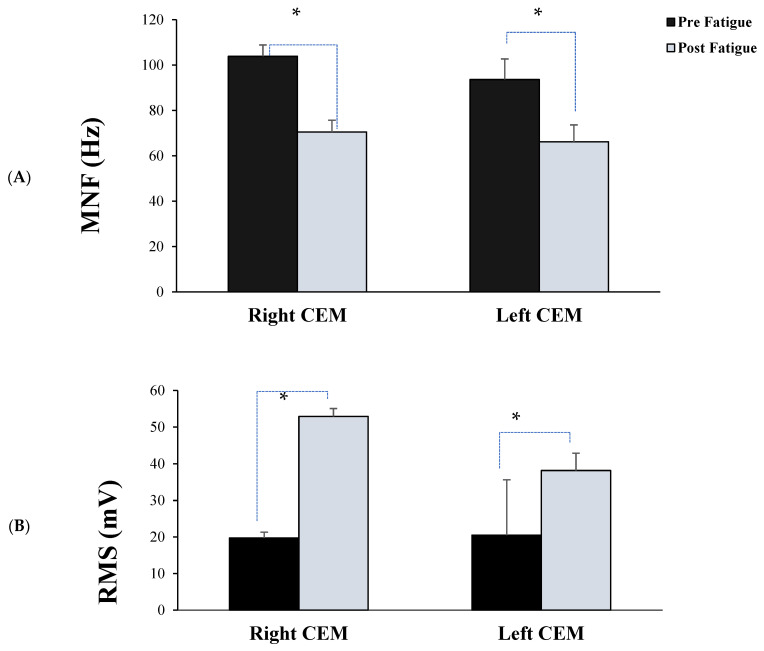
Average (**A**) mean power frequency (MNF), and (**B**) root mean square (RMS) for both right and left CEM during the pre and post fatigue protocol for fatigue group. Significant differences between pre and post fatigue existed for both the MNF and RMS. Error bars represent SD. * *p* < 0.001.

Post-fatigue MNF was significantly lower than baseline for both right and left CEM (F_1,22_ = 9.65, *p* = 0.0001) (Figure 4A), with no significant interaction between right and left CEM (F_1,22_ = 0.547, *p* = 0.374). Post-fatigue RMS amplitude was significantly greater than baseline for both right and left CEM (F_1,22_ = 10.968, *p* = 0.003) (Figure 4B), with no significant interaction between right and left CEM (F_1,22_ = 1.026, *p* = 0.322]).

### 5.3. Behavioral Data

**Figure 5:**
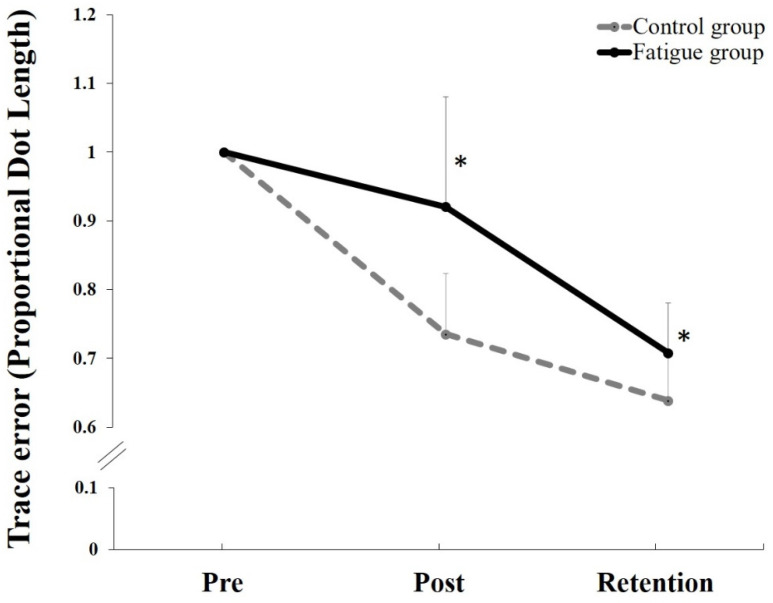
Normalized mean proportional trace error changes by groups (Control vs. Fatigue). Both groups improved in accuracy after motor skill acquisition tracing tasks and during the retention (*p* < 0.001). The control group outperformed the fatigue group in both post-motor skill acquisition and during retention (* *p* < 0.05). Error bars represent SD.

**Figure 6:**
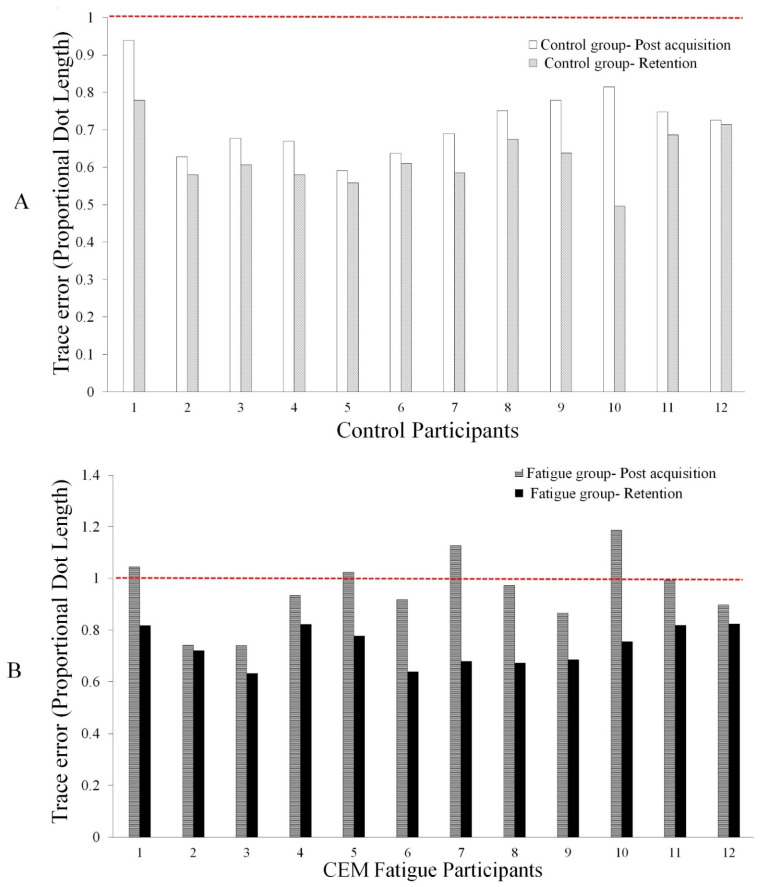
Changes in motor performance post-acquisition and retention for (**A**) control, and (**B**) CEM fatigue groups. Individual performance change shown relative to normalized pre acquisition performance (dotted red line). The control group demonstrated a greater improvement post acquisition (*p* = 0.0001) and at retention (*p* = 0.002) than the fatigue group.

There was a significant main effect of TIME (F_1,22_ = 114.82, *p* < 0.0001, partial eta squared (η^2^) = 0.839) and a significant interaction of TIME by GROUP (F_1,22_ = 15.239, *p* = 0.0001, η^2^ = 0.409) for the normalized motor skill acquisition tracing data. A post hoc pairwise comparison using the Bonferroni correction showed significant increase in accuracy for both groups following motor skill acquisition (*p* = 0.0001) and at retention (*p* = 0.0001), with the control group demonstrated a greater improvement after both motor skill acquisition (F_1,22_ = 23.55, *p* = 0.0001, η^2^ = 0.517) and at retention (F_1,22_ = 12.78, *p* = 0.002, η^2^ = 0.368), (27% and 37%), as compared to the fatigue group (4% and 26%), respectively (Figure 5 and Figure 6).

### 5.4. Neurophysiological SEPs Data

The inclusion criteria for SEP data analysis was that the N9 SEP peak recorded over the brachial plexus, should differ by no more than ±20% between pre and post intervention trials in order for that participant’s data to be included based on IFCN guidelines [40]. This is to ensure that positional changes in the electrode to the underlying nerve did not affect the afferent volley. Although care was taken when repositioning the participant for SEP recording after the fatigue and/or control intervention, the N9 was not stable for some participants and the results for 2 control and 2 fatigue group participants could not be further analyzed as they failed to meet the inclusion criteria. In total, 20 participants were included in the analysis of SEP peaks (10 control and 10 fatigue). SEPs results are reported as (mean percent ± SD).

**Figure 7:**
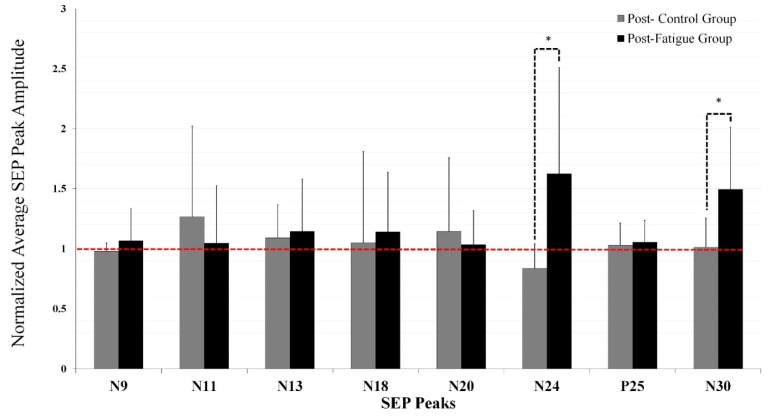
Averaged normalized SEP Peak amplitude ratios relative to baseline (dotted line) showing control vs. fatigue groups following motor skill acquisition tracing task. Note: significant interactive effects between groups were found for N24 and N30 SEP peaks (* *p* < 0.05). Following motor skill acquisition tracing task, significant changes from baseline were observed for both the control and fatigue group on N24 SEP peak and for fatigue group on N30 SEP peak. Error bars represent SD.

**Figure 8:**
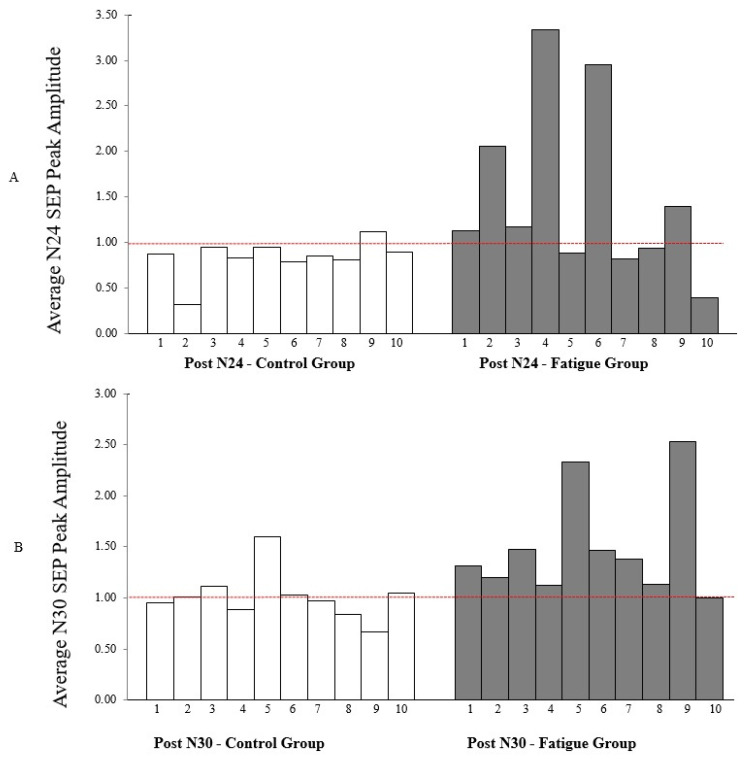
Graphical Representation of each individual performance, (**A**) N24 SEP peak, and (**B**) N30 SEP peak time traces (difference per participants between pre- and post-acquisition). The N24 did not increase in any of the control participants, but it did in the majority of the CEM neck fatigue participants. With the N30, only one control participant showed an increased SEP amplitude, whereas nine of the ten CEM neck fatigue participants showed an increase, while the other participant showed no change.

N24 SEP peak: Following motor skill acquisition, there was a significant interaction of TIME by GROUP [F_1,18_ = 4.576, *p* = 0.046, η^2^ = 0.203] with no effect of TIME (*p* = 0.287). The N24 decreased by 16.4 ± 20% for the control group and increased by 62.34 ± 88% for the fatigue group. (Figure 7 and Figure 8). The achieved statistical power (1-β error prob) for the time by group effect size η^2^ = 0.39426 (f = 0.6955) with an alpha of 0.05 was 0.999 [57].

N30 SEP peak: Following motor skill acquisition, there was a significant TIME effect (F_1,18_ = 7.888, *p* = 0.012, η^2^ = 0.305), and a significant interaction of TIME by GROUP (F_1,18_ = 7.101, *p* = 0.016, η^2^ = 0.283). The control group N30 amplitude was 1.2 ± 24% after motor skill acquisition (*p* = 0.879), while the fatigue group increased by 49 ± 51% (F_1,9_ = 9.114, *p* = 0.015, η^2^ = 0.503) (Figure 7 and Figure 8). The achieved statistical power (1-β error prob) for the time by group effect size η^2^ = 0.394 (f = 0.8063) and an alpha of 0.05 was 0.999 [57].

**Figure 9:**
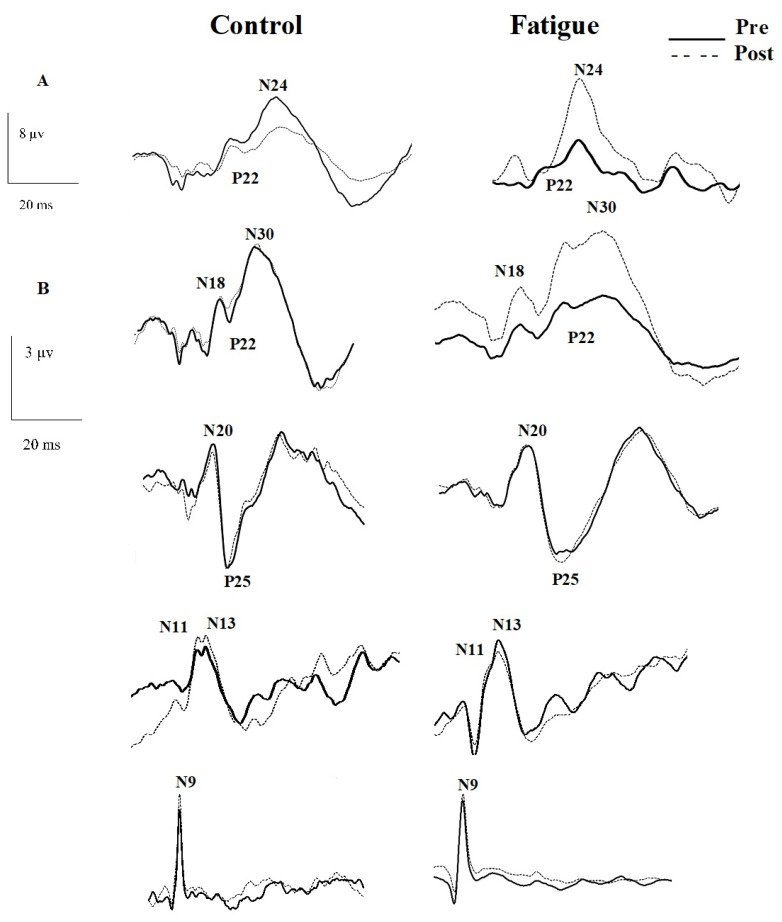
Raw data from a representative control and fatigue participant indicating cortical SEP peak amplitudes. (**A**) Fast rate (2.47 Hz), (**B**) Slow rate (4.98 Hz).

There was no significant difference between groups for N11 (F_1,18_ = 0.353, *p* = 0.560, η^2^ = 0.019), N13 (F_1,18_ = 0.006, *p* = 0.937, η^2^ = 0.0004), N18 (F_1,18_ = 0.003, *p* = 0.954, η^2^ = 0.0002), N20 (F_1,18_ = 0.519, *p* = 0.481, η^2^ = 0.028), and P25 (F_1,18_ = 0.676, *p* = 0.422, η^2^ = 0.036), (Figure 7, Figure 8 and Figure 9).

## 6. Discussion

This study investigated the interactive effect of CEM fatigue and motor skill acquisition on sensorimotor processing by measuring changes in early SEP peak amplitudes between control and fatigue groups. In keeping with hypothesis one, differential changes were evoked in early SEP peaks related to SMI in the CEM fatigue vs. control group following motor skill acquisition. The amplitude of the N24 SEP peak significantly decreased for the control group and increased for the fatigue group. In addition, the N30 SEP peak amplitude significantly increased for the fatigue group and not the control group. There were significant improvements in accuracy for both groups, suggestive that motor learning occurred. However, in line with hypothesis two, we observed significantly less performance accuracy both post motor skill acquisition and at subsequent task retention following fatigue, as compared to the control group. Neck muscle fatigue has been shown to alter upper limb motor performance [16] and chronic changes in neck input alters cerebellar processing in response to motor learning [2]. This study is the first to report differential SEP peak amplitude changes (in frontal N24 and N30), in response to acute alterations in neck sensory input induced by fatigue.

Muscle fatigue activates nociceptors by metabolic products of muscle contraction including bradykinin, arachidonic acid, prostaglandin E2, potassium, and lactic acid, with these metabolites increasing the threshold for muscle spindle discharge and subsequently changes alpha– gamma co-activation [58]. In addition, muscle fatigue increases automatic release in mechanically sensitive non-spindle group II and group III muscle afferents [59]. Changes in muscle spindle discharge alter afferent feedback, and subsequently alter conscious joint awareness [60]. Isometric muscle contraction requires voluntary neural drive from the motor cortex that is accompanied by increased fusimotor drive, with in response more muscle spindles is recruited, resulting in a stronger sensory illusion [61]. Therefore, altered somatosensory input from the neck due to fatigue could result in alterations in central neural processing of the altered sensory input, as demonstrated by SEP peak amplitude changes. Past work found that the cerebellum to motor cortex pathway was more inhibited when neck fatigue preceded motor acquisition [28]. In fact, the neck fatigue group showed significantly less disinhibition than the non-fatigued control group.

### 6.1. N24 SEP Peak Changes

In the current study, the tracing task leads to differential changes between the control and CEM fatigue groups. The decreased N24 SEP peak in the control group is consistent with previous studies that found decreased N24 SEP amplitudes following a simple automatic repetitive typing task [52] and after a motor learning tracing task, similar to the one used in the current study [2]. In a previous study, the N24 SEP peak showed a larger decrease for the tracing task in comparison to the repetitive typing task, likely due to fact that these sorts of tracing tasks rely heavily on cerebellar pathways [21]. The N24 SEP peak is recorded from the contralateral frontal cephalic site. The N24 SEP peak reflects activation of neurons in the pathway between the cerebellum- primary sensory area (SI) and is thus likely to reflect changes in cerebellar output [33]. Therefore, the decreased N24 amplitude in the control participants of the current study is likely related to decrease cerebellar nuclei activity [7], which was also associated with the greater improvement in motor skill acquisition. This is also in line with previous transcranial magnetic stimulation (TMS) studies that showed a decrease in cerebellar inhibition following motor skill acquisition [62] and previous functional magnetic resonance imaging (fMRI) studies which found that disinhibition of cerebellar output is reflective of the decreased reliance on cerebellar activity when a task is well learned [7,21]. 

The increase in the N24 in the fatigue group in the current study is consistent with a previous study by Andrew et al. (2018) that investigated chronic changes in neck sensory input in a neck pain population following a motor training task. Their work also found an increased N24 SEP peak in response to motor skill acquisition, for neck pain participants vs. a healthy control group, which may reflect increased cerebellar-SI processing in this group. The neck pain participants did not reach the later stage of learning in the same way as the healthy control group [2]. The SEP changes in peaks related to cerebellar processing indicate that the altered sensory input from the neck to the CNS likely impacted the expected neuroplastic changes in cerebellar pathways in response to motor skill acquisition [2].

The differential N24 SEP peak changes may also reflect the role of the cerebellum in sensorimotor prediction [63] and SMI [64]. Differences between predicted and real movements are used by the CNS to update feed forward models, and an accurate model is critical for accurate motor performance [65]. The cerebellum is a fundamental integrator of sensory information that is critical for the development of internal models when learning complex motor tasks [66]. The cerebellum is known to contribute to sensorimotor integration, error correction, and also the development of internal models [22]. Internal models are used to predict the relationship between motor commands and the predicted sensory consequences of that movement [22], allowing comparison between predicted and actual consequences of an action. Integration of this error signal by the Purkinje cells enables motor learning, by providing the CNS with an error signal that can be used to correct subsequent movements. Internal models can be modified via comparison between motor plans generated in the motor cortex, and the sensory feedback generated by the actual movement conveyed via the spinocerebellar pathway in the inferior olive, which contains the climbing fiber inputs responsible for the error signal to the Purkinje cell. This internal model then updates the link between the motor commands and the expected sensory consequences. Therefore, any changes in this cerebellar input due to altered sensory feedback from the periphery can alter the information from the cerebellum to the motor cortex motor cortex, potentially leading to maladaptive plasticity [22].

Given that the cerebellum is an important integration site for motor learning [21,67] as well as for SMI of the sensory input from the joints of the neck and spine [64,68], altered neck input is likely to have impacted the accuracy of the internal body schema. Information about muscle activity is delivered to the somatosensory cortex through afferent feedback from muscle, tendons, and joints. This information is processed by the cerebellum, which then sends output to motor areas so that efferent motor commands to the limbs are adjusted accordingly, to generate a more accurate movement [69,70]. The cerebellum receives and incorporates sensory input prior to modifying output through Purkinje cells which output to cortical regions [71]. These enable the learning of smooth, constant motions and the formation of an accurate internal body schema [71] thus permitting learners to progressively adjust and decrease errors in motor skill acquisition performance [64]. These studies align with a previous TMS study by our group, which showed that neck fatigue impacted the cerebellar “disinhibition” that normally accompanies the early stage of learning of a novel task [28].

fMRI studies have demonstrated that the cerebellum is activated during both the early and later stages of motor learning [72]. The regional blood flow in the cerebellum increases in the early stage of motor skill acquisition, and then decreases as performance improves [73]. Interestingly, the activity of a specific sub-region of the cerebellum, near the posterior superior fissure, remained activated even once participants had learned a novel task, which is thought to represent an area where an acquired internal model is stored [74]. The decrease in the N24 SEP amplitude in the control group of the current study may reflect a decrease in cerebellar nuclei activity, which is related to the later stages of learning, reflecting a greater reliance on internal body schema, with new encodings that reflect the post learning state [21]. In contrast, the CEM fatigue group had increased cerebellar-SI processing, confirmed by an increased N24 SEP amplitude. This suggests that the fatigue group did not reach the later stage of learning in the same way as the control group. In support of these findings, previous studies have shown that in order to learn a novel task and correct performance error, when a task is not well-learned the activity of certain parts of the brain including the cerebellum increases, while once a task is well-learned, these “error detection” areas do not need to be as active [7,64].

### 6.2. N30 SEP Peak Changes

The N30 peak is a complex subcortical and cortical loop connecting the basal ganglia, thalamus, pre-motor areas, primary motor cortex (MI) [31,32], supplementary motor area (SMA) [75], and reflects the overarching process of SMI [33]. Dipole source localization found that sources in the cingulate cortex, bilateral secondary somatosensory cortex (SII), contralateral primary somatosensory cortex (SI), and prefrontal cortex all contributed to the N30 SEP peak [76]. The N30 SEP peak component is known as a critical index of brain sensorimotor processing. It has been shown that it goes along with an increase amplitude of the constant beta-gamma rhythm peaking at 30 Hz [77,78]. 

In the current study, the motor skill acquisition tracing task leads to an increased N30 SEP peak for both groups. The fatigue group had a greater increase compared to the control group, which is in line with previous studies [2,79]. The greater N30 SEP peak in the fatigue group post- motor skill acquisition may reflect attempts by the SMI integration network [2] to integrate the altered neck muscle sensory feedback following fatigue [16], resulting in altered excitability in pathways relevant to motor learning.

### 6.3. Behavioral Data

Significant improvements in accuracy were observed for both groups (decreased error), suggesting that motor learning had occurred for both the control and CEM fatigue groups. However, overall accuracy differed by group, with the control group outperforming the fatigue group following both motor skill acquisition and retention. The better motor skill acquisition accuracy for the control group is in line with previous studies that investigated the impact of motor learning, using the same tracing task, and found significant improvements in accuracy observed after both acquisition and retention [2,9]. The worse performance by the fatigue group indicates that the altered neck input has impaired upper limb motor skill acquisition. It is noteworthy that the differential N24 and N30 SEP changes coincide with worse motor performance in the neck fatigue group.

Motor skill can be improved by using motor prediction, mental practice, or sensorimotor plasticity induced through movement performance [66]. The CNS implements prediction by using internal models to simulate the interaction between the body and external environment [80]. Internal models are neural representations to estimate how, for example, the arm moves in response to a motor command, relative to the environment, and its own current position and velocity, which is known as an internal forward model [81,82]. In a previous target tracking study, Selen et al. (2007) fatigued the elbow flexor and extensor muscles and participants were required to track a sinusoidal moving target using elbow flexion and extension [83]. Post fatigue, the participants changed their control strategy to a feedforward plan, staying closer to the middle of the target than in the unfatigued state [83]. In this study, neck fatigue, may have resulted in an altered body schema subsequent to altered neck sensory input, which would force increased reliance on feedforward processes. 

### 6.4. Effect of Fatigue

We used the same CEM fatigue protocol in a previous TMS study [28]. In that study, we collected EMG activities of the middle and lower trapezius muscle to determine if additional muscles other than cervical extensor muscles contributed to the neck muscle fatigability. That study found that MNF of the middle trapezius increased from the beginning to the end of the fatigue protocol and it did not change for lower trapezius [28]. The increase in MNF in the middle trapezius indicates that rather than fatiguing, the middle trapezius was beginning to increase its activation levels, most likely to compensate for the fatiguing CEM. This data indicated that the fatigue task is able to specifically fatigue the CEM. Previously the same CEM fatigue protocol led to increased cerebellar inhibition of the primary motor cortex, and CEM fatigue prior to motor skill acquisition task led to a decreased capacity for cerebellar disinhibition in response to the motor acquisition task. This suggests that CEM fatigue, may affect the cerebellar-motor cortex interaction, influencing the cerebellum’s ability to adjust motor output to perform a novel motor skill acquisition task with greater accuracy [28]. In addition, in another study the same tracing task as was used in the current study, was compared to simple mental recitation, and SEP changes were only seen after the motor tracing task, with no changes in response to the mental arithmetic task [84], indicating the differential SEP changes in the current study are specific to the impact of the altered neck input due to fatigue on the neural response to motor learning.

In the current study, there was a significant decrease in force from the pre to the post MVCs, confirming that neck muscle fatigue occurred [85]. During sustained submaximal, low-intensity muscle contractions over time, a decrease in force production occurs due to changes in both facilitatory and inhibitory influences at the nerve endings (axonal terminals) and at the neuromuscular junction (NMJ) [86]. The neuromuscular system attempts to compensate for the decrease in force generation and delays declines in force production [87]. Decreases in force generation are usually associated with both central and peripheral fatigue [27]. Central fatigue can account for the drop in sustained force productions [27], due to a decline in motor cortex excitation and a decrease in motoneuron activity [88]. The differential SEP changes in the CEM fatigue group in response to motor learning indicates that neck fatigue has the ability to impact upper limb motor performance and skill acquisition learning. It is clear that fatigue resulted in SEP peak differences relative to the control group, interfering with the usual response to motor skill acquisition.

Branscheidt et al. (2019) [89] assessed how muscle fatigue influenced skill learning over multiple days. In this study, both fatigue and non-fatigue participants learned a sequential isometric pinch force task over two days by holding the force transducer between the thumb and index finger of their dominant hand. They were instructed to decrease and increase their pinching force at different force levels to navigate the motion of a cursor displayed on the screen, through the four different gates. The fatigue group was only fatigued at day one and they were instructed to press the force transducer with their maximum until the force dropped to the greatest extent (~ 60% decrement of MVC) and always stayed above the force level required for task performance (up to 40% of MVC). Time to fatigue was ~ 70 s on average. To counterbalance the amount of time of the fatigue induction, the non-fatigue group was sustained at 5% of their MVC with the same muscle group over a matched period without experiencing force decline. On day two, both groups performed the motor skill task without induction of muscle fatigue. 

Results showed that for both groups the ability to execute the task was improved on both days with lower learning rate for the fatigue group. Muscle fatigue resulted in lower levels of performance immediately after fatigue. On day two, the fatigue group did not even reach the same performance level that the non-fatigue participants had at the end of day one regardless of having double the amount of training and in the absence of fatigue on day two. Importantly, there were no differences in learning rate within either the fatigue or non-fatigue group (day 1 versus day 2), with learning rates remaining low in the fatigue group, and achieving only 68% of the performance level of the non-fatigue group. These results show that motor learning under fatigue conditions has a long-lasting negative effect on motor skill acquisition on subsequent practice days even in the absence of fatigue. Both groups took two additional days (day 3 and day 4) of training identical to day 2, to assess how much practice the fatigue group took to have a similar performance level as the non-fatigue group. The negative effect of fatigue continued for almost two complete additional training sessions; only towards the end of day 3 and day 4 did the fatigue group catch up to the performance level of the non-fatigued group. These results demonstrate that muscle fatigue has overall long- lasting adverse effects on motor skill learning.

From our current study, the amount of CEM fatigue may be different from the beginning to the end of the motor skill acquisition task. However, the negative effect of the fatigue on motor learning in fatigue group is evident in the impaired task performance on the retention day, which occurred in the absence of fatigue. CEM fatigue has overall long-term effects on motor skill learning, suggesting that the muscle fatigue lasted long enough to impact skill acquisition and subsequent retention.

## 7. Limitations

One potential limitation is that the contributions of the vestibular system to an internal body schema for upper limb motor tasks could be partially responsible for the observed differences, but given that the head was in the identical position for the control group (e.g., both groups were in a prone position for the fatigue or control portion), and seated during task performance, this is unlikely to be the main contributor to the observed differences. Additionally, given that neck muscle vibration, which activates muscles spindles, influences upper limb proprioception, even though the head is held in the same position, it is clear that altered neck muscle input has the capacity to alter upper limb proprioception and motor control.

Another potential limitation is that we used a fixed load. Although we had clear neurophysiological and performance differences due to fatigue with this fixed load, it is possible that if we had used a relative load based on the MVC or strength of the participants, it might have resulted in even bigger differences between the groups.

It should be noted that while the fatigue participants showed less improvement at acquisition, four fatigue participants did not improve at all at acquisition. Nonetheless, all four showed improvement at retention, indicating that they really did try to learn the task and were just worse at acquisition following neck fatigue.

While single EEG electrodes on the scalp is appropriate for recording short latency SEP peaks changes in small hand muscles (e.g., median nerve carrying feedback from thenar muscles of thumb), the results of this study indicate the need for whole head EEG recordings to assess whole-arm tasks. The N30 SEP peak has multiple neural generators, and a whole head EEG system would create the ability to do source localization of the N30 in order to understand which neural generators are contributing to the observed difference following fatigue.

## 8. Conclusions

This study suggests that CEM fatigue influences how the CNS learns an upper limb motor task. This work demonstrated that the cerebellum may be particularly affected by CEM fatigue, as well as SMI pathways, as indicated by the differential changes in the magnitude of the N24 and N30 SEP peak amplitudes following motor learning.

The worse performance accuracy in the CEM fatigue group indicates that input from the neck affects upper limb movement performance. An important direction for future work is to investigate the role and mechanism of the cerebellum as well as SMI integration areas in the response to neuromuscular fatigue of the neck muscles. As the acquisition of new upper limb motor skills often occurs in conjunction with neck fatigue in a variety of occupational and recreational settings, the influence of CEM muscular fatigue on motor learning and neuroplasticity is critical to understand, to ensure that that the neuroplasticity associated with motor learning is adaptive and not maladaptive.

## Figures and Tables

**Table 1:** Participant demographics and self-report measures.

	Control Group	Fatigue Group	*t*-Test Alpha Results
	(6 Females-6 Males)	(6 Females-6 Males)	
	Mean (SD)	Mean (SD)	
Age	20.91 (0.7)	21.58 (0.9)	*t* (11) = −2, *p* = 0.071
NDI score	1.75 (1.6)	2.16 (1.6)	*t* (11) = −0.613, *p* = 0.552
EHI score	63.33 (23.1)	78.33 (18.5)	*t* (11) = −2.22, *p* = 0.062
Handedness	12 right handed	12 right handed	
Time to task failure (min)	5 min neck muscle rest	Average: 6.29 (2.8) Female: 5.82 (2.0) Male: 6.76 (3.6)	*t* (11) = 0.805, *p* = 0.457 No male–female difference

## Data Availability

All of the individual data for SEP peaks was included in Figure 7 and Figure 8.
